# Repeated Quantitative Fetal Fibronectin Assessments for Preterm Birth Prediction in Pregnant Women with Cervical Insufficiency

**DOI:** 10.3390/diagnostics16162667

**Published:** 2026-08-21

**Authors:** Fanny Mikula, Ricarda Heemann, Anika Schoberwalter, Katharina Goeral, Alex Farr, Hanns Helmer, Stephanie Springer

**Affiliations:** 1 Department of Obstetrics and Gynecology, Division of Obstetrics and Feto-Maternal Medicine, Comprehensive Center for Pediatrics, Medical University of Vienna, 1090 Vienna, Austria; fanny.mikula@meduniwien.ac.at (F.M.);; 2 Department of Pediatrics and Adolescent Medicine, Division of Neonatology, Pediatric Intensive Care and Neuropediatrics, Comprehensive Center for Pediatrics, Medical University of Vienna, 1090 Vienna, Austria

**Keywords:** cervical insufficiency, preterm birth, quantitative fetal fibronectin, QUiPP App

## Abstract

**Background/Objectives**: Preterm birth remains the leading cause of neonatal morbidity and mortality, with cervical insufficiency being a major risk factor. Quantitative fetal fibronectin (qfFN) and the QUiPP App version 2.0 are established tools for preterm birth prediction; however, the value of repeated assessments remains unclear. This study aimed to evaluate the prognostic accuracy of longitudinal qfFN measurements and QUiPP risk assessment in women with progressive cervical insufficiency and to evaluate the prognostic performance of repeated assessments. **Methods:** In this retrospective cohort study, 88 women with progressive cervical insufficiency who underwent repeated qfFN measurements were included. Cervical length, qfFN, and QUiPP risk were assessed at diagnosis and at subsequent time points. Prognostic performance for delivery within one week and before 34 and 37 gestational weeks was evaluated. **Results:** The prognostic performance represented by the area under the curve increased from 0.71 to 0.75 for qfFN and from 0.74 to 0.82 for QUiPP from the first to the final assessment, although neither difference reached statistical significance (qfFN *p* = 0.522; QUiPP risk *p* = 0.135). For imminent preterm birth, qfFN demonstrated excellent rule-out performance, with a negative predictive value of 100% at <10 ng/mL. QUiPP risk provided superior risk stratification and achieved excellent prognostic accuracy for delivery before 34 weeks at the final assessment (area under the curve = 0.82). Receiver operating characteristic analysis identified optimal cutoffs of approximately 40 ng/mL for qfFN and 23% for QUiPP risk in predicting delivery before 34 weeks. **Conclusions:** Repeated qfFN assessment combined with QUiPP risk estimation was associated with numerically higher discriminatory performance at the final assessment. While the identified optimal cutoff values need to be validated in prospective trials, they might have the potential to safely reduce unnecessary interventions in the future. qfFN functioned as an effective rule-out test, whereas QUiPP enhanced risk stratification, supporting their combined repeated use to guide clinical management before 34 + 0 weeks of gestation.

## 1. Introduction

Preterm birth (PTB) remains the leading cause of neonatal mortality worldwide [1]. PTB is classified as extremely preterm (before 28 + 0 weeks of gestation), very preterm (28 + 0 to 31 + 6 weeks), and moderate-to-late preterm (32 + 0 to 36 + 6 weeks) [2]. Neonatal morbidity and mortality increase disproportionately with decreasing gestational age, particularly below 34 weeks, where the risks of respiratory distress syndrome, intraventricular hemorrhage, necrotizing enterocolitis, and long-term neurodevelopmental impairment rise substantially [3]. With a global prevalence of approximately 10%, PTB affects an estimated 13.4 million newborns annually [4]. Among the multiple contributing factors, cervical insufficiency, defined as a cervical length < 25 mm before 34 + 0 weeks, represents a major risk factor for PTB [5,6]. Clinical management depends on presentation and gestational age and may include antenatal corticosteroids, tocolysis, progesterone, and magnesium sulfate [5,7]. Accurate prognostic assessment is essential to guide appropriate treatment, particularly because the beneficial effect of fetal lung maturation is limited to approximately 10 days [8]. Fetal fibronectin (fFN), a glycoprotein located at the utero-placental interface, is among the most established biomarkers for predicting PTB [9]. Typically barely present in cervicovaginal secretions, fFN is released in response to mechanical stress or inflammatory processes [10]. Between 22 and 34 weeks of gestation, qualitative detection of FN (commonly interpreted as positive > 50 and negative < 50 ng/mL) aids in assessing the risk of PTB within the subsequent week [11,12]. With a negative predictive value (NPV) of up to 99.5%, it helps to avoid unnecessary interventions [13]. Quantitative fFN (qfFN) allows more refined risk stratification [14,15]. Levels < 10 ng/mL are associated with a very low risk, whereas levels > 100 ng/mL are linked to a high risk of PTB before 34 weeks, particularly in asymptomatic women with a short cervix [16,17]. In the context of cervical insufficiency, qfFN improves risk stratification by providing higher sensitivity and superior NPV for PTB compared with cervical length measurements alone [11,18]. By integrating qfFN values with a history of PTB, prior cervical surgery, cervical length, and gestational age, the QUiPP App generates an individualized estimate of PTB within one week and before 34 and 37 gestational weeks [19]. Therefore, it serves as a practical decision-support tool in clinical care [20]. Although the predictive value of a single qfFN measurement in high-risk populations is well established, data on longitudinal qfFN trends during pregnancy and their clinical relevance remain limited. A prospective study in a low-risk cohort demonstrated that increasing qfFN concentrations between 20 and 300 ng/mL at 24–30 weeks were associated with an elevated risk of PTB [21]. However, comparable data for high-risk populations, including women with cervical insufficiency, are lacking.

Despite limited evidence regarding temporal changes in qfFN in women with cervical insufficiency, repeated measurements are frequently performed in clinical practice to help guide clinical management in case of progressive cervical insufficiency. Therefore, this study evaluates the prognostic value of repeated measurements of cervical length, qfFN, and QUiPP risk in pregnancies complicated by progressive cervical insufficiency, aiming to evaluate whether predictive accuracy differs between initial and repeated assessments and thereby inform clinical management.

## 2. Materials and Methods

### 2.1. Ethics Statement

This retrospective cohort study was conducted in accordance with the STROBE checklist for cohort studies [22], the Declaration of Helsinki, and Good Scientific Practice guidelines. The study protocol was approved by the local ethics committee (application number: 1284/2024).

### 2.2. Study Design and Clinical Management

Pregnant women with cervical insufficiency who were treated at the Department of Obstetrics and Gynecology, Division of Obstetrics and Feto-Maternal Medicine, Medical University of Vienna, between June 2021 and December 2024 were included. Cervical insufficiency was diagnosed by transvaginal sonography in accordance with locally applicable guidelines [5]. It was defined as a cervical length ≤ 25 mm before 34 + 0 weeks of gestation [5]. The examination was performed with an empty urinary bladder and visualization of the cervix along its entire longitudinal axis, occupying 50–75% of the screen diameter. At least three measurements were obtained without applying pressure, and the shortest measurement guided further management. Sonographic examinations were routinely performed using sterile probe covers without external gel application. After diagnosis, vaginal swabs were collected from the posterior fornix during a sterile speculum examination for qfFN measurement (Rapid fFN Test, Hologic Inc., Marlborough, MA, USA). Based on these findings and patients’ history, the QUiPP risk (version 2.0 2017) was calculated [17,18,19,20].

All women with cervical insufficiency received routine vaginal supplementation with micronized progesterone. Women presenting with signs of preterm labor received tocolysis for 48 h. When the QUiPP risk for PTB within one week exceeded 5%, two doses of betamethasone were administered for fetal lung maturation, with concomitant tocolysis for 48 h. If new symptoms occurred, such as lower abdominal pain or progressive cervical shortening before 34 + 0 weeks, the initial examination, including sonography and qfFN measurement with QUiPP risk calculation, was repeated. Repeated testing was performed in women with progressive cervical insufficiency, defined as new symptoms suggestive of preterm labor and/or progressive cervical shortening during follow-up.

Women were excluded for contraindications to qfFN measurement (e.g., vaginal bleeding, sexual intercourse within the previous 24 h, prior ultrasound with external gel application, rupture of membranes, or cervical dilation of >3 cm) [23], multiple gestation, cervical cerclage, age < 18 years, missing repeated qfFN measurements, or iatrogenic PTB. Demographic and perinatal data were extracted from the ViewPoint Fetal Database (version 5.6.28.56; General Electric Company, GE Viewpoint, Munich, Germany) and the institutional hospital information system. To ensure data accuracy, two independent investigators monitored the data collection process.

### 2.3. Statistical Analysis

For statistical analyses, the time of cervical insufficiency diagnosis, corresponding to the initial assessment of cervical length and qfFN, was defined as the first time point. The final combined measurement during pregnancy was defined as the final time point. The prognostic performance of cervical length, qfFN, and QUiPP risk estimates was evaluated using receiver operating characteristic (ROC) curve analysis, with the area under the curve (AUC) quantifying discrimination for PTB at different gestational time points. DeLong’s test for correlated ROC curves was used to perform paired comparisons of the ROC curves obtained at the first and final assessments. Due to clinical relevance, cervical length, qfFN, and QUiPP risk estimates were evaluated separately, although it needs to be acknowledged that they are mathematically related variables. Given its therapeutic relevance, delivery before 34 + 0 weeks was defined as the primary outcome. Data for delivery within one week as well as before 37 weeks were analyzed as secondary outcomes. These time points derive from the calculated risk scores within the QUiPP App, as well as their relevance in clinical decision-making. Sensitivity, specificity, positive predictive value (PPV), and NPV were calculated at clinically relevant thresholds to assess rule-in and rule-out performances. Optimal cut-offs for qfFN and QUiPP risk were determined using the Youden index (J), defined as the maximum sum of sensitivity and specificity, to identify thresholds with the highest overall diagnostic accuracy. For endpoints with low event rates, previously established cut-offs were applied to reduce the risk of overfitting. Changes in cervical length, qfFN, and QUiPP risk between the first and final time points were additionally analyzed to assess their predictive value. All analyses were two-sided, and 95% confidence intervals were calculated for diagnostic performance measures where appropriate. Complete data were available for all variables included in the primary analyses. No imputation was required, and all analyses were performed using complete-case data.

## 3. Results

### 3.1. Patient Cohort

A total of 88 women with cervical insufficiency and at least two qfFN measurements were included. Cervical insufficiency was first diagnosed at 24 + 6 weeks of gestation (interquartile range [IQR]: 23 + 0 to 27 + 0), which constituted the first time point for cervical length and qfFN assessment. The final assessment was conducted at 29 + 6 weeks (IQR: 27 + 0 to 32 + 4). The median cervical length at diagnosis was 15.5 mm (IQR: 10–21), and the median qfFN concentration was 35 ng/mL (IQR: 10–164). Sixteen women (18.2%) had a history of previous PTB. Baseline demographic characteristics are presented in Table 1.

### 3.2. Gestational Age at Delivery and Neonatal Outcomes

Overall, 42 of the 88 women (47.7%) experienced PTB. Among these, 27 (64.3% of PTBs) delivered before 34 + 0 weeks. The median gestational age at delivery was 37 + 2 weeks (IQR: 32 + 3 to 39 + 0), and the median birth weight was 2883 g (IQR: 1781–3380). One intrauterine fetal demise occurred; all other births were live deliveries. Admission to the neonatal intensive care unit was required for 31 neonates (35.2%), all of whom survived to discharge. Delivery and neonatal outcomes are summarized in Table 2.

### 3.3. Prognosis Prediction

At the first time point, cervical length demonstrated acceptable discrimination for delivery before 34 weeks (AUC = 0.76) and poor discrimination for delivery before 37 weeks (AUC = 0.67). qfFN showed comparable performance, with acceptable discrimination for delivery before 34 weeks (AUC = 0.71) but poor discrimination for delivery before 37 weeks (AUC = 0.63). The QUiPP risk demonstrated acceptable discrimination for delivery before 34 weeks (AUC = 0.74) and before 37 weeks (AUC = 0.71). No analysis was performed for delivery within one week of the first time point because only one woman delivered during this interval.

**Figure A1:**
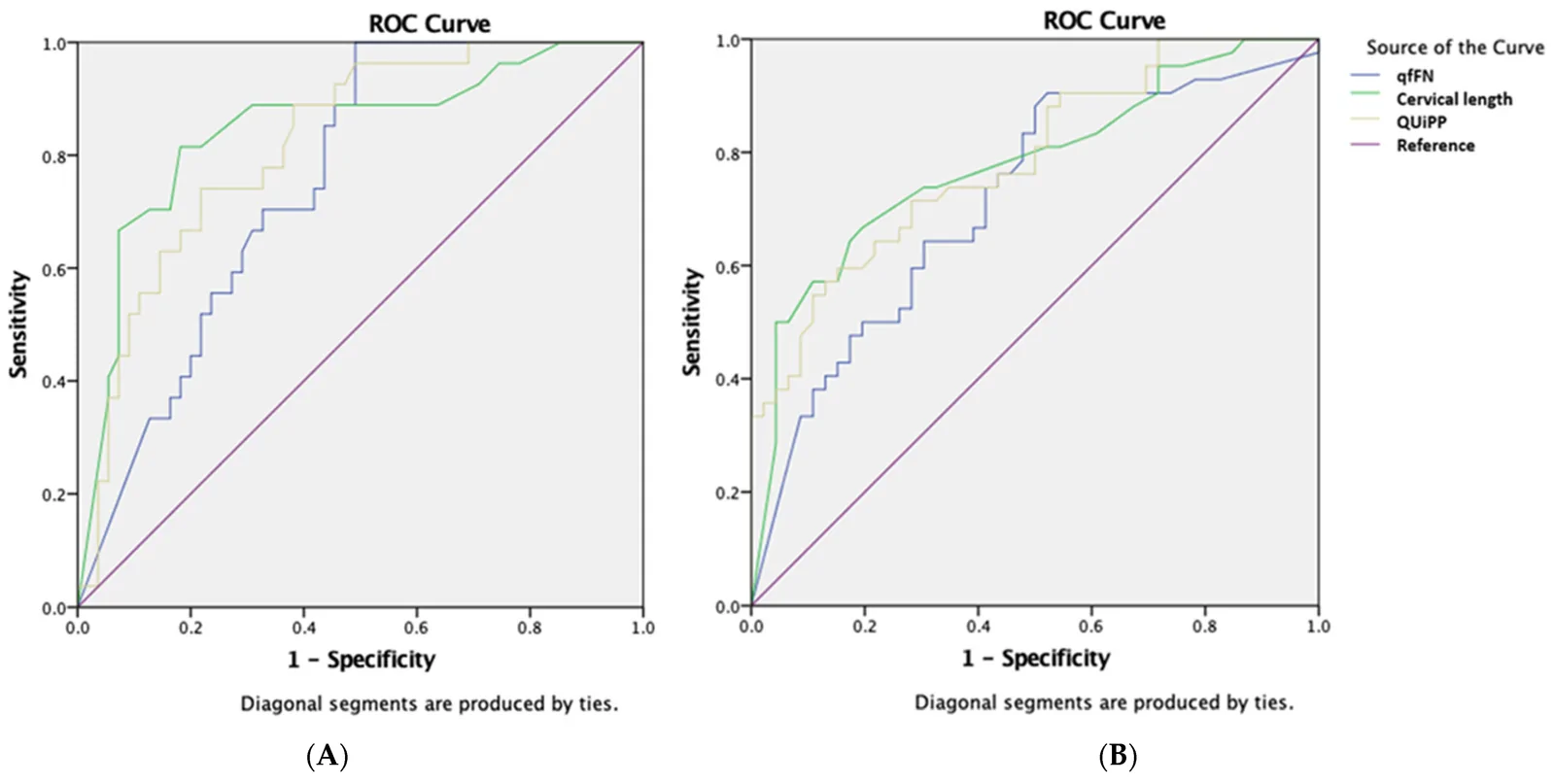
Receiver operating characteristic curves at the final assessment for (**A**) delivery before 34 weeks and (**B**) delivery before 37 weeks. The purple reference lines represent an area under the curve of 0.5.

At the final time point, cervical length showed poor discrimination for delivery within one week (AUC = 0.69), excellent discrimination for delivery before 34 weeks (AUC = 0.84), and acceptable discrimination for delivery before 37 weeks (AUC = 0.78). qfFN demonstrated acceptable discrimination for delivery within one week, before 34 weeks, and before 37 weeks (AUC = 0.75, 0.75, and 0.72, respectively). The corresponding QUiPP risk provided acceptable discrimination for delivery within one week (AUC = 0.72) and before 37 weeks (AUC = 0.79) and excellent discrimination for delivery before 34 weeks (AUC = 0.82). These results are summarized in Table 3, and the corresponding ROC curves are shown in Figure A1. Although numerically higher AUCs were observed at the final assessment for all evaluated predictors, none of the differences reached statistical significance for prediction of delivery before 34 weeks (qfFN *p* = 0.522; cervical length *p* = 0.166; QUiPP risk *p* = 0.135) or before 37 weeks (qfFN *p* = 0.223; cervical length *p* = 0.076; QUiPP risk *p* = 0.174).

Changes between the first and final time points were additionally analyzed. Cervical length decreased by a median of 3.5 mm (IQR: −9 to 0.75), demonstrating poor discrimination for delivery before 34 and 37 weeks (AUC = 0.61 for both outcomes). qfFN increased in 53 women (60.2%), remained stable in 3 (3.4%), and decreased in 32 (36.4%), with a median relative increase of 39.8% (IQR: −48.7 to 791.9). These changes demonstrated poor to very poor discrimination for delivery before 34 and 37 weeks (AUC = 0.60 and 0.59, respectively). The QUiPP risk for delivery before 34 weeks changed by a median of −1.3% (IQR: −12.2% to 15.6%), yielding poor discrimination (AUC = 0.65). The QUiPP risk for delivery before 37 weeks increased slightly by 1.1% (IQR: −8% to 25.1%) and also demonstrated poor discrimination (AUC = 0.60). Detailed results are provided in Table A1.

### 3.4. Risk Stratification

To enhance the clinical relevance of these findings, rule-in and rule-out performances were evaluated for the final time point, given the numerically higher AUCs observed at the final assessment. For delivery within one week, previously established cut-offs for cervical length, qfFN, and QUiPP risk were applied [10,16,19,24] because the low event rate (*n* = 88; 12 events) rendered ROC-based cut-off optimization statistically unstable and prone to overfitting. As shown in Table 4, qfFN demonstrated excellent rule-out performance. Using a cut-off of 10 ng/mL, no woman with qfFN < 10 ng/mL delivered within 7 days (NPV: 100%, 95% CI: 84.6–100), whereas 12 of 66 women with qfFN ≥ 10 ng/mL delivered within 7 days (PPV: 18.2%, 95% CI: 10.0–29.5). Application of the conventional 50 ng/mL cut-off [10] yielded a similar PPV (19.3%, 95% CI: 10.1–31.9) with a slightly lower NPV (96.8%, 95% CI: 83.3–99.9). QUiPP provided improved risk stratification, with PPV increasing from 18.2% (95% CI: 9.1–30.9) at a 5% cut-off to 22.5% (95% CI: 11.8–37.5) at a 10% cut-off.

Given the higher event rate for delivery before 34 weeks (*n* = 88; 27 events), ROC-based optimization of cut-offs was performed for qfFN and QUiPP risk, whereas cervical length was not subjected to ROC-derived threshold optimization because clinically established cut-offs are lacking and the study primarily focused on qfFN and QUiPP thresholds (Table 4). ROC analysis identified an optimal qfFN cut-off of approximately 40 ng/mL (J = 0.509) and an optimal QUiPP risk cut-off of approximately 23% (J = 0.507). Detailed calculations are provided in Table A2. Results for delivery before 37 weeks are not presented because of their limited relevance for preventive interventions and only acceptable, rather than superior, discriminatory performance.

## 4. Discussion

Our data demonstrate that discriminatory performance was numerically higher at later assessments compared to the initial assessment, particularly when qfFN testing was combined with repeated application of the QUiPP App, even though these findings did not reach statistical significance. Temporal changes in qfFN or QUiPP risk did not enhance predictive performance; however, the final QUiPP risk estimate for delivery before 34 + 0 weeks provided the highest prognostic accuracy. Cervical length and QUiPP demonstrated the highest discriminatory performance for delivery before 34 weeks, whereas qfFN alone demonstrated superior rule-out performance, and QUiPP provided the strongest rule-in capability and overall risk stratification.

Although cervical length assessment is readily available, and a cervical length of <25 mm before 34 + 0 weeks is a validated risk factor for PTB, its predictive performance varied according to the timing of assessment. At the initial evaluation, cervical length provided acceptable discrimination for delivery before 34 weeks (AUC 0.76) and poor discrimination for delivery before 37 weeks (AUC 0.67). At the final assessment, however, cervical length demonstrated excellent discrimination for delivery before 34 weeks (AUC 0.84) and acceptable discrimination for delivery before 37 weeks (AUC 0.78). These findings suggest that cervical length remains an important predictor in women with cervical insufficiency, particularly when measured closer to delivery [25].

Substantial research has focused on identifying additional biomarkers to improve PTB prediction in women with cervical insufficiency, with vaginal fFN being among the most established. Although qualitative testing is widely used for PTB prediction [11], quantitative measurement provides greater prognostic accuracy [14]. To further enhance predictive performance, the QUiPP App was developed to integrate qfFN with clinical parameters [17,18,19,20]. In this cohort, cervical length and QUiPP risk demonstrated the highest discriminatory performance for delivery before 34 weeks at the final assessment (AUC 0.84 and 0.82, respectively), both yielding numerically higher AUCs than qfFN alone. However, QUiPP additionally provides integrated risk stratification by combining cervical length, qfFN, gestational age, and obstetric history into a single clinically applicable risk estimate. In contrast, trajectory-based changes added no predictive value beyond absolute measurements. Using established cut-offs to predict delivery within 7 days of the final sampling time point, qfFN demonstrated excellent rule-out performance. With an NPV of 96–100% and a PPV of 18–19%, qfFN primarily functioned as a rule-out test for imminent PTB, with particularly strong negative predictive performance close to delivery. In comparison, QUiPP offered improved risk stratification, with a PPV ranging from 18% to 23% and an NPV of 94%. For delivery before 34 weeks, ROC analysis identified an optimal qfFN cut-off of approximately 40 ng/mL, compared with the commonly used 50 ng/mL threshold [10], providing the best balance between sensitivity and specificity. Similarly, the optimal QUiPP cut-off for PTB before 34 weeks was 23%, which is substantially higher than the commonly applied 5% or 10% thresholds [19,24].

Although the QUiPP App is typically applied at cervical insufficiency diagnosis to guide decisions regarding antenatal corticosteroid administration, it remains unclear whether repeated assessment in the setting of progressive cervical shortening improves predictive accuracy. Our results demonstrate that the diagnostic performance of the QUiPP App was higher at later assessments compared to the initial assessment, particularly for predicting PTB before 34 weeks, with the AUC increasing from 0.74 to 0.82, consistent with excellent discrimination.

Our findings indicate that cervical length, qfFN, and the QUiPP App provide complementary tools rather than competing tests for PTB prediction. While cervical length and QUiPP demonstrated the strongest overall discrimination for delivery before 34 weeks, qfFN provided the best rule-out performance for imminent PTB, whereas QUiPP offered the most clinically useful integrated risk stratification. Although the commonly applied qfFN cut-off was similar to the optimal threshold identified in our analysis, the optimal QUiPP cut-off was substantially higher than previously described values. Given that the QUiPP App was originally evaluated against a treat-all strategy [19], emphasis was placed on safety, particularly on strong rule-out performance to ensure that interventions such as antenatal corticosteroids can be safely withheld. Our findings raise the hypothesis that higher QUiPP risk estimates (up to approximately 23% in this cohort) might still permit safe withholding of interventions when qfFN levels are low (<40 ng/mL in this cohort), given the robust rule-out performance of qfFN. If these values could be validated in larger prospective trials, this might be a particularly relevant finding for women with a markedly shortened cervix and an unfavorable obstetric history. However, ROC-derived optimal cut-offs reflect internal performance within this cohort and should not be interpreted as universally applicable clinical thresholds but rather as hypothesis-generating data for future studies.

Repeated assessment was associated with higher AUCs for qfFN and QUiPP risk, particularly for predicting PTB before 34 weeks. Given that imminent PTB before 34 weeks necessitates specific interventions to improve neonatal outcomes, such as antenatal corticosteroid administration, these findings are clinically relevant. A similar time-dependent increase in AUCs was observed for PTB before 37 weeks; however, discriminatory performance remained acceptable rather than excellent. This may reflect the longer interval between assessment and delivery and greater population heterogeneity. Although late PTB remains clinically relevant, it typically does not require specific preventive interventions, thereby limiting the clinical impact of predictive performance within this gestational window.

Consistent with the excellent discriminatory performance of the final QUiPP risk estimate and the numerically higher discriminatory performance observed at the final assessment, our findings suggest potential clinical utility of repeated qfFN testing with corresponding QUiPP risk calculation for women with progressive cervical insufficiency before 34 + 0 weeks. However, the absence of statistically significant AUC differences and the inability to separate repeated testing from assessment timing preclude conclusions regarding incremental predictive benefit.

These findings suggest that qfFN and QUiPP risk constitute a complementary combination with strong rule-out and rule-in capabilities. Although established cut-offs remain appropriate for qfFN as a rule-out test, the Youden index analysis identified a substantially higher optimal QUiPP cut-off than commonly applied thresholds [10,19,24]. Evaluation of this higher threshold in therapeutic decision-making, specifically when combined with a negative qfFN result, represents an important area for future research and may reduce unnecessary interventions. Given the retrospective design and limited sample size, external validation of these findings, particularly the proposed optimal cut-offs, is warranted in adequately powered prospective trials.

This study has both strengths and limitations. Including only women who underwent repeated qfFN testing limits the generalizability of the findings to high-risk cases with progressive cervical insufficiency. The relatively small cohort likely reflects the limited number of women undergoing repeated qfFN testing and may also have limited the precision and statistical power of comparisons between assessments, highlighting the need for confirmation in larger prospective trials. In addition, the numerically higher discriminatory performance observed at the final assessment should be interpreted with caution. Although paired DeLong tests showed no statistically significant differences between the first and final AUCs, the final assessments were performed later in pregnancy and closer to delivery than the initial assessments, and the numerically higher AUCs may therefore partly reflect temporal proximity to the outcome rather than an independent benefit of serial testing itself. Moreover, repeated assessments were clinically triggered rather than performed at predefined gestational-age intervals, making assessment timing itself informative. Although gestational age at assessment was available, simple covariate adjustment would not fully account for this time-dependent selection process. Adjustment for time-to-delivery was not appropriate because this information is only known after delivery and is therefore not available at the time of prediction. The present dataset therefore did not permit a robust assessment of incremental predictive value independent of assessment timing. Prospective studies with predefined testing intervals or appropriately powered dynamic prediction approaches are required to address this question. Because QUiPP risk estimates were incorporated into clinical decision-making, including the administration of antenatal corticosteroids and tocolysis at a threshold >5%, some degree of management bias cannot be excluded. This may have attenuated or altered the observed relationship between QUiPP risk and subsequent delivery outcomes. Furthermore, direct comparisons between QUiPP and its individual components should be interpreted cautiously, as cervical length and qfFN are incorporated into the QUiPP algorithm. Finally, the retrospective, single-center design may limit the generalizability of the findings to other populations and management protocols. However, the application of robust statistical methods, including ROC and AUC analyses to quantify discriminatory performance and Youden index calculations to identify optimal cut-offs, strengthens the validity of the results. To our knowledge, this is the first study to evaluate longitudinal qfFN testing in women with cervical insufficiency.

## 5. Conclusions

Quantitative fFN demonstrates acceptable prognostic accuracy for predicting PTB, which can be enhanced by integrating clinical parameters through QUiPP risk calculation. Later assessment demonstrated numerically higher discriminatory performance, particularly for the primary endpoint of delivery before 34 + 0 weeks, for which the final QUiPP estimate achieved excellent discriminatory accuracy. qfFN provides superior rule-out capability, whereas QUiPP offers enhanced risk stratification. Our findings support consideration of repeated combined qfFN testing and QUiPP risk calculation within diagnostic algorithms for women with progressive cervical insufficiency before 34 + 0 weeks to optimize clinical decision-making.

## Figures and Tables

**Table 1:** Baseline demographic and obstetric characteristics of women with cervical insufficiency.

Characteristics	Participants, *n* = 88
Age, years (median [IQR])	33 (29–36)
Body mass index, kg/m^2^ (median [IQR])	23.9 (21.3–27.7)
Gravidity (median [IQR])	2 (1–4)
Parity (median [IQR])	1 (1–2)
Spontaneous conception (abs. + rel. frequency)	70 (79.5%)
Previous conization (abs. + rel. frequency)	8 (9.1%)
Smoking during pregnancy (abs. + rel. frequency)	6 (6.9%)
Diabetes mellitus (abs. + rel. frequency)	19 (21.6%)
Hypertensive disorder (abs. + rel. frequency)	9 (10.2%)
Estimated fetal weight < 10th centile (abs. + rel. frequency)	4 (4.5%)
Vaginal bleeding during pregnancy (abs. + rel. frequency)	22 (25%)
Vulvovaginal infection (abs. + rel. frequency)	23 (26.1%)
Previous early pregnancy loss (abs. + rel. frequency)	31 (35.2%)
Previous late pregnancy loss (abs. + rel. frequency)	14 (15.9%)
Previous preterm birth (abs. + rel. frequency)	16 (18.2%)

IQR = interquartile range; abs. = absolute; rel. = relative.

**Table 2:** Risk parameters, obstetric management, delivery data, and neonatal outcomes in the study cohort, which included women with cervical insufficiency.

Characteristic	Participants, *n* = 88
Cervical length	
Cervical length at first time point, mm (median [IQR])	15.5 (10–21)
Cervical length at final time point, mm (median [IQR])	10 (4.2–16)
Quantitative Fetal Fibronectin	
qfFN at first time point, ng/mL (median [IQR])	35 (10–164)
qfFN at final time point, ng/mL (median [IQR])	142.5 (10–441.5)
QUiPP Risk	
QUiPP risk < 7 days at first time point, %	0.9 (0.3–3.3)
QUiPP risk < 34 weeks at first time point, %	26.7 (12.8–45.8)
QUiPP risk < 37 weeks at first time point, %	47.1 (27–66.8)
QUiPP risk < 7 days at final time point, %	9.1 (2.8–21.7)
QUiPP risk < 34 weeks at final time point, %	24.9 (12.4–69)
QUiPP risk < 37 weeks at final time point, %	57.7 (28.3–84.9)
Obstetric Complications and Management	
Preterm labor < 34 weeks (abs. + rel. frequency)	46 (52.3%)
pPROM < 34 weeks (abs. + rel. frequency)	13 (14.8%)
Antenatal corticosteroid administration (abs. + rel. frequency)	61 (69.3%)
Neonatal Outcomes	
Live birth (abs. + rel. frequency)	87 (98.9%)
Preterm birth < 37 weeks (abs. + rel. frequency)	42 (47.7%)
Preterm birth < 34 weeks (abs. + rel. frequency)	27 (32.9%)
Gestational age at delivery (median [IQR])	37 + 2 (32 + 3 to 39 + 0)
Fetal weight at delivery, g (median [IQR])	2883 (1781–3380)
Apgar score at 5 min (median [IQR])	10 (9–10)
Umbilical cord pH (median [IQR])	7.29 (7.21–7.34)
NICU admission, days (median [IQR])	0 (0–13)

abs. = absolute; rel. = relative; pPROM = preterm premature rupture of membranes; IQR = interquartile range; NICU = neonatal intensive care unit; qfFN = quantitative fetal fibronectin. The first time point (defined in the Section 2) occurred at 24 + 6 weeks (IQR: 23 + 0 to 27 + 0), and the final time point occurred at 29 + 6 weeks (IQR: 27 + 0 to 32 + 4).

**Table 3:** AUC estimates based on ROC analyses for qfFN, cervical length, and QUiPP risk in predicting delivery within 1 week and before 34 and 37 weeks.

	Delivery Within 1 Week	Delivery Before 34 Weeks	Delivery Before 37 Weeks
First Time Point			
Cervical length		0.76	0.67
qfFN		0.71	0.63
QUiPP risk < 34 weeks		0.74	
QUiPP risk < 37 weeks			0.71
Final Time Point			
Cervical length	0.69	0.84	0.78
qfFN	0.75	0.75	0.72
QUiPP risk (1 week)	0.72		
QUiPP risk < 34 weeks		0.82	
QUiPP risk < 37 weeks			0.79
≥0.9 = outstanding			
0.89–0.8 = excellent			
0.79–0.7 = acceptable			
0.69–0.6 = poor			
0.59–0.5 = very poor			
<0.5 = no discrimination			

qfFN = quantitative fetal fibronectin. The first time point (defined in the Section 2) occurred at 24 + 6 weeks (IQR: 23 + 0 to 27 + 0), and the final time point was at 29 + 6 weeks (IQR: 27 + 0 to 32 + 4). Only one woman (1.1%) delivered within 1 week of the first assessment; therefore, ROC analysis was not performed for this outcome because the AUC estimate would have been statistically unstable and lacked interpretive precision.

**Table 4:** Diagnostic accuracy measures at established cutoffs for cervical length, qfFN, and QUiPP risk for predicting delivery within 1 week at the final time point.

Test	Cut-Off	Sensitivity (95% CI)	Specificity (95% CI)	PPV (95% CI)	NPV (95% CI)
Cervix	15 mm	16.7% (2.1–48.4)	69.7% (58.0–79.7)	8.0% (1.0–26.0)	84.1% (72.6–92.1)
qfFN	10 ng/mL	**100% (73.5**–**100)**	28.9% (19.0–40.5)	18.2% (10.0–29.5)	**100% (84.6**–**100)**
qfFN	50 ng/mL	91.7% (61.5–99.8)	39.5% (28.3–51.6)	19.3% (10.1–31.9)	96.8% (83.3–99.9)
QUiPP	5%	83.3% (51.6–97.9)	40.8% (29.6–52.8)	18.2% (9.1–30.9)	93.9% (79.8–99.3)
QUiPP	10%	75.0% (42.8–94.5)	**59.2% (47.3**–**70.4)**	**22.5% (11.8**–**37.5)**	93.8% (82.8–98.7)

CI = confidence interval; PPV = positive predictive value; NPV = negative predictive value; qfFN = quantitative fetal fibronectin. The bold numbers indicate the numerically highest finding for each category within the primarily evaluated parameters qfFN and QUiPP risk.

## Data Availability

The data analyzed during the current study are not publicly available due to patient confidentiality and institutional restrictions but are available from the corresponding author upon reasonable request, subject to approval by the institutional review board and in compliance with applicable data protection regulations.

## Abbreviations

The following abbreviations are used in this manuscript:

PTB	Preterm Birth
qfFN	Quantitative Fibronectin
AUC	Area under the Curve
ROC	Receiver operating characteristic
NPV	Negative predictive value
PPV	Positive predictive value
IQR	Interquartile range

## Appendix A

**Table A1:** AUC estimates based on ROC analyses for changes in cervical length, qfFN, and QUiPP risk between the first and last assessments for predicting delivery before 34 + 0 and 37 + 0 weeks.

	Delivery < 34 Weeks	Delivery < 37 Weeks
Δ Cervical length	0.61	0.61
Δ qfFN	0.6	0.59
Δ QUiPP risk < 34 weeks	0.65	
Δ QUiPP risk < 37 weeks		0.6
≥0.9 = outstanding		
0.89–0.8 = excellent		
0.79–0.7 = acceptable		
0.69–0.6 = poor		
0.59–0.5 = very poor		
<0.5 = no discrimination		

qfFN = quantitative fetal fibronectin. The first time point (defined in the Section 2) occurred at 24 + 6 weeks (IQR: 23 + 0 to 27 + 0), and the final time point occurred at 29 + 6 weeks (IQR: 27 + 0 to 32 + 4).

**Table A2:** Youden index analysis identifying the optimal cut-off values for predicting delivery before 34 weeks based on qfFN and QUiPP risk. Optimal cut-off values are shown in bold.

	Sensitivity	Specificity	Youden Index (J)
**Cut-off qfFN**			
25.5	1.000	0.491	0.491
39.0	**1.000**	**0.509**	**0.509**
51.0	0.963	0.509	0.472
59.0	0.889	0.509	0.398
63.0	0.889	0.527	0.416
68.0	0.889	0.545	0.434
72.0	0.852	0.545	0.397
81.0	0.852	0.564	0.416
100	0.815	0.564	0.379
**Cutoff QUiPP**			
10.25	1.000	0.273	0.273
12.00	0.963	0.327	0.290
13.40	0.963	0.418	0.381
14.90	0.963	0.473	0.436
16.15	0.963	0.509	0.472
16.45	0.926	0.527	0.453
18.05	0.926	0.545	0.471
19.45	0.889	0.545	0.434
20.30	0.889	0.564	0.453
22.55	0.889	0.600	0.489
23.30	**0.889**	**0.618**	**0.507**
23.60	0.852	0.618	0.470
23.80	0.815	0.636	0.451

qfFN = quantitative fetal fibronectin; J = Sensitivity + Specificity − 1.
